# Effect of physical exercise training in patients with Chagas heart disease: study protocol for a randomized controlled trial (PEACH study)

**DOI:** 10.1186/s13063-016-1553-4

**Published:** 2016-09-02

**Authors:** Fernanda de Souza Nogueira Sardinha Mendes, Andréa Silvestre Sousa, Fernando Cesar de Castro Cesar Souza, Vivian Liane Mattos Pinto, Paula Simplicio Silva, Roberto Magalhães Saraiva, Sergio Salles Xavier, Henrique Horta Veloso, Marcelo Teixeira Holanda, Andréa Rodrigues Costa, Fernanda Martins Carneiro, Gilberto Marcelo Sperandio Silva, Juliana Pereira Borges, Eduardo Tibirica, Roberta Olmo Pinheiro, Flávio Alves Lara, Alejandro Marcel Hasslocher-Moreno, Pedro Emmanuel Alvarenga Americano Brasil, Mauro Felippe Felix Mediano

**Affiliations:** 1 Evandro Chagas National Institute of Infectious Diseases, Oswaldo Cruz Foundation, Avenida Brasil 4365, Manguinhos, Rio de Janeiro 21040-360 Brazil; 2 National Institute of Cardiology, Rua das Laranjeiras 374, Laranjeiras, Rio de Janeiro 22240-006 Brazil; 3 Physical Education and Sports Institute, State University of Rio de Janeiro, Rua São Francisco Xavier, 524, Maracanã, Rio de Janeiro 20550-900 Brazil; 4 Oswaldo Cruz Institute, Oswaldo Cruz Foundation, Avenida Brasil 4365, Manguinhos, Pavilhão Cardoso Fontes, Sala 64, Rio de Janeiro, 21040-360 Brazil

**Keywords:** Chagas heart disease, Heart failure, Exercise training, Cardiac rehabilitation, Cardiopulmonary exercise test

## Abstract

**Background:**

The effects of exercise training on Chagas heart disease are still unclear. This study aimed to evaluate the effect of exercise training over functional capacity, cardiac function, quality of life, and biomarkers in Chagas heart disease.

**Methods:**

The PEACH study is a superiority randomized clinical trial which will include subjects who meet the following criteria: Chagas heart disease with a left ventricular ejection fraction below 45 % with or without heart failure symptoms; clinical stability in the last 3 months; adherence to clinical treatment; and age above 18 years. The exclusion criteria are: pregnancy; neuromuscular limitations; smoking; evidence of non-chagasic heart disease; systemic conditions that limit exercise practice or cardiopulmonary exercise test; unavailability to attend the center three times a week during the intervention period; and practitioners of regular exercise. The intervention group will perform an exercise training intervention three times per week during 6 months and will be compared to the control group without exercise. Both groups will undergo the same monthly pharmaceutical and nutritional counseling as well as standard medical treatment according to the Brazilian consensus on Chagas disease. The primary outcome is functional capacity based on peak exercise oxygen consumption during cardiopulmonary exercise testing. Secondary outcomes are: cardiac function; body composition; muscle respiratory strength; microvascular reactivity; cardiac rhythm abnormalities; autonomic function; biochemical; oxidative stress and inflammatory biomarkers; and quality of life. Subjects will be evaluated at baseline, and at 3 and 6 months after randomization. Thirty patients will be randomly assigned into exercise or control groups at a ratio of 1:1.

**Discussion:**

Findings of the present study will be useful to determine if physical exercise programs should be included as an important additional therapy in the treatment of patients with Chagas heart disease.

**Trial registration:**

ClinicalTrials.gov ID: NCT02517632 (registered on 6 August 2015).

**Electronic supplementary material:**

The online version of this article (doi:10.1186/s13063-016-1553-4) contains supplementary material, which is available to authorized users.

## Background

Chagas heart disease (CHD) is the most common manifestation of chronic Chagas disease with prevalence of 20 to 30 % in patients infected with *Trypanosoma cruzi* [1]. Patients with CHD present a high incidence of cardiac complications, morbidity, and mortality in Latin America [2]. The Brazilian consensus on Chagas disease [3] classifies CHD into different stages that reflect prognosis: stage A (abnormalities on electrocardiogram attributable to Chagas disease and no left ventricular (LV) wall motion abnormalities detected by echocardiography), stage B1 (LV wall motion abnormalities with an ejection fraction (EF) >45 % and no heart failure (HF)), stage B2 (LVEF <45 % and no HF), stage C (compensated HF), and stage D (endstage HF).

Currently, CHD therapy is based on treating symptoms and slowing the heart disease progression following the standard guidelines for cardiac disease of other etiologies [4]. However, some particular features in the clinical course of CHD demand specific treatment. Therefore, studies evaluating the effects of different strategies on patients with CHD are necessary.

Treatment of heart disease requires a multidisciplinary team-based care approach that includes exercise training to improve patients’ functional status [5]. Cardiac rehabilitation (CR) is associated with consistent improvements in symptoms, cardiac mortality, number of hospitalizations, quality of life, and in numerous relevant clinical endpoints [6]. Moreover, exercise programs have gained increased recognition during the past years and have been strongly recommended by many different cardiology societies in the world, mainly for non-CHD [7–9].

However, exercise studies including patients with Chagas disease are warranted since these patients are usually not included in exercise clinical trials [10]. The first study that addressed the effects of exercise training on CHD showed that functional capacity, clinical symptoms, and some domains of health-related quality of life (vitality, emotional aspects, and mental health) improved after 3 months of follow-up [11]. Another single-arm study demonstrated that oxygen consumption at peak of exercise (VO_2_ peak), oxygen pulse (O_2_ pulse), and oxygen consumption at anaerobic threshold (VO_2_ AT) improved after 6 months of exercise training [12]. However, the interpretation of these results is limited by the short-term follow-up in the former study and the lack of a control group in the later study. Thus, new well-designed clinical trials are necessary to improve the knowledge of CR effects on patients with CHD.

The PEACH study, which stands for “Exercise Program in Chagas Heart Disease” in Portuguese, is designed to address the effects of exercise training in patients with CHD. The primary aim of the study is to investigate the effects of exercise on functional capacity in patients with CHD, measured as the VO_2_ peak during a maximal progressive cardiopulmonary exercise test (CPET). Secondary objectives are to assess the effects of exercise training on cardiac function, body composition, muscle respiratory strength, microvascular reactivity, cardiac rhythm abnormalities, autonomic function, biochemical, inflammatory and oxidative stress biomarkers, and quality of life. We hypothesized that exercise training will be safe and will promote important clinical benefits mainly on functional capacity among patients with CHD.

## Methods

### Study design

The PEACH study is a single-center, superiority randomized clinical trial (ClinicalTrials.gov ID: NCT02517632) performed at the Evandro Chagas National Institute of Infectious Diseases (INI), a national reference center for treatment and research in infectious and tropical diseases in Rio de Janeiro, Brazil. The unit staff is composed of infectious disease specialists, cardiologists, gastroenterologists, nurses, pharmacists, and exercise physiologists. Resources such as echocardiography, computed tomography, digestive endoscopy, 24-h Holter electrocardiogram monitoring, ambulatory blood pressure monitoring, CPET, and cardiac rehabilitation are also available. The institute has outpatient and inpatient treatment sectors with an intensive care unit.

### Participants and recruitment

Individuals followed at INI will be sequentially recruited to participate in the study. The eligibility criteria are: (1) Chagas disease diagnosis, confirmed by two simultaneous serological tests (enzyme-linked immunosorbent assay (ELISA) and indirect immunofluorescence) [3], (2) CHD with LVEF <45 % with or without HF symptoms at baseline evaluation (CHD stages B2 and C), (3) New York Heart Association class I or II in the previous 3 months, with clinical stability by investigator judgment, (4) clinical treatment according to HF guidelines, including treatment with angiotensin-converting enzyme inhibitors or angiotensin receptor blockers, and beta-blocker therapy, or documented rationale for variation, including intolerance, contraindication, or patient preference [13, 14]. Patients will be on stable doses of medications for 6 weeks prior to enrollment, and (5) age above 18 years. Exclusion criteria are: (1) pregnancy, (2) neuromuscular limitations that preclude physical exercise, (3) smoking, (4) evidence of known non-chagasic heart disease, (5) systemic conditions that limit exercise practice or CPET, (6) unavailability to attend the center three times a week during the intervention period, and (7) practitioners of regular exercise.

**Fig. 1:**
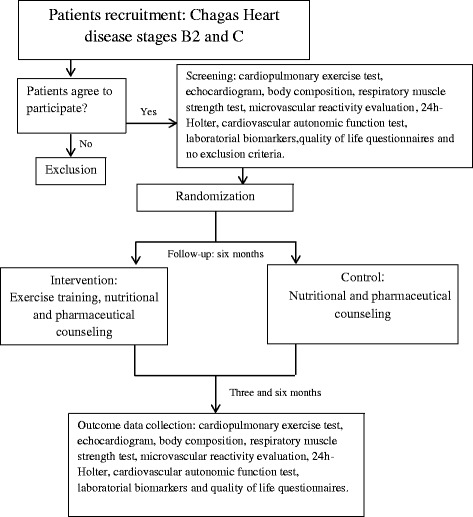
Flow diagram of the PEACH study protocol

Trained research assistants or study investigators will conduct the evaluations in a quiet and private room. Initial interviews will include a questionnaire to collect sociodemographic data (income, race, age, sex, and schooling), medical history (stage of CHD, comorbidities, medication, cardiac devices, and arrhythmias), functional class, and level of physical activity through the International Physical Activity Questionnaire-Short Form (IPAQ-SF). Evaluations of functional capacity (maximal progressive CPET), cardiac function (two-dimensional echocardiography), body composition (anthropometry and skinfolds), muscle respiratory strength (manovacuometry), microvascular reactivity (laser speckle flowmetry), cardiac rhythm abnormalities (24-h Holter), autonomic function (active orthostatic stress test), laboratorial biomarkers (biochemical, inflammatory, and oxidative stress), and quality of life (Short Form-36 (SF36) and Minnesota Living with Heart Failure Questionnaire) will be performed at baseline, after 3 months, and at the end of follow-up (6 months). After initial evaluation, patients will be randomized to intervention or control groups as seen in Fig. 1.

### Intervention

Patients included in the intervention arm will be submitted to an exercise training performed three times a week for 60 min, during a 6-month period. Each session will comprise 30 min of aerobic exercise on a treadmill or cycle ergometer (the first 5 min of warm-up and the last 5 min of cool-down), 20 min of strength exercises for the major muscle groups (sit-ups, push-ups, and pull-ups), and 10 min of stretching. The exercise intensity will be set according to the heart rate obtained during the CPET, corresponding to the anaerobic threshold minus 10 % in the first month of exercise protocol and the anaerobic threshold plus 10 % in the following months. Blood pressure, heart rate, and oxygen saturation will be measured before, during aerobic exercise (at 20 min of aerobic exercise), and at the end of each training session using an aneroid sphygmomanometer (Unitec, São Paulo, Brazil), a heart rate monitor (Polar FT1, Kempele, Finland), and an oximeter (IMFtec®, São Paulo, Brazil). Individuals with long QT syndrome, atrial fibrillation or flutter with ventricular response less than 100 bpm at rest, frequent premature ventricular contractions, sinus tachycardia, sinus bradycardia, or patients with any cardiac related-symptoms will have their rhythm observed with a cardiac monitor during the exercise sessions. Glucose monitoring will be performed before and after exercise sessions in diabetic patients to guarantee its safety. For pre-exercise blood glucose levels of less than 100 mg.dL^−1^ (5.5 mmol.L^−1^), the American Diabetes Association recommends that carbohydrate should be ingested before any exercise. The patient will be able to engage in exercise with levels exceeding 300 mg.dL^−1^ (16.7 mmol.L^−1^) unless they present with ketosis symptoms, or dehydration [15]. All training sessions will be performed in the morning, indoors, and with a controlled temperature environment under supervision of medical staff.

**Fig. 2:**
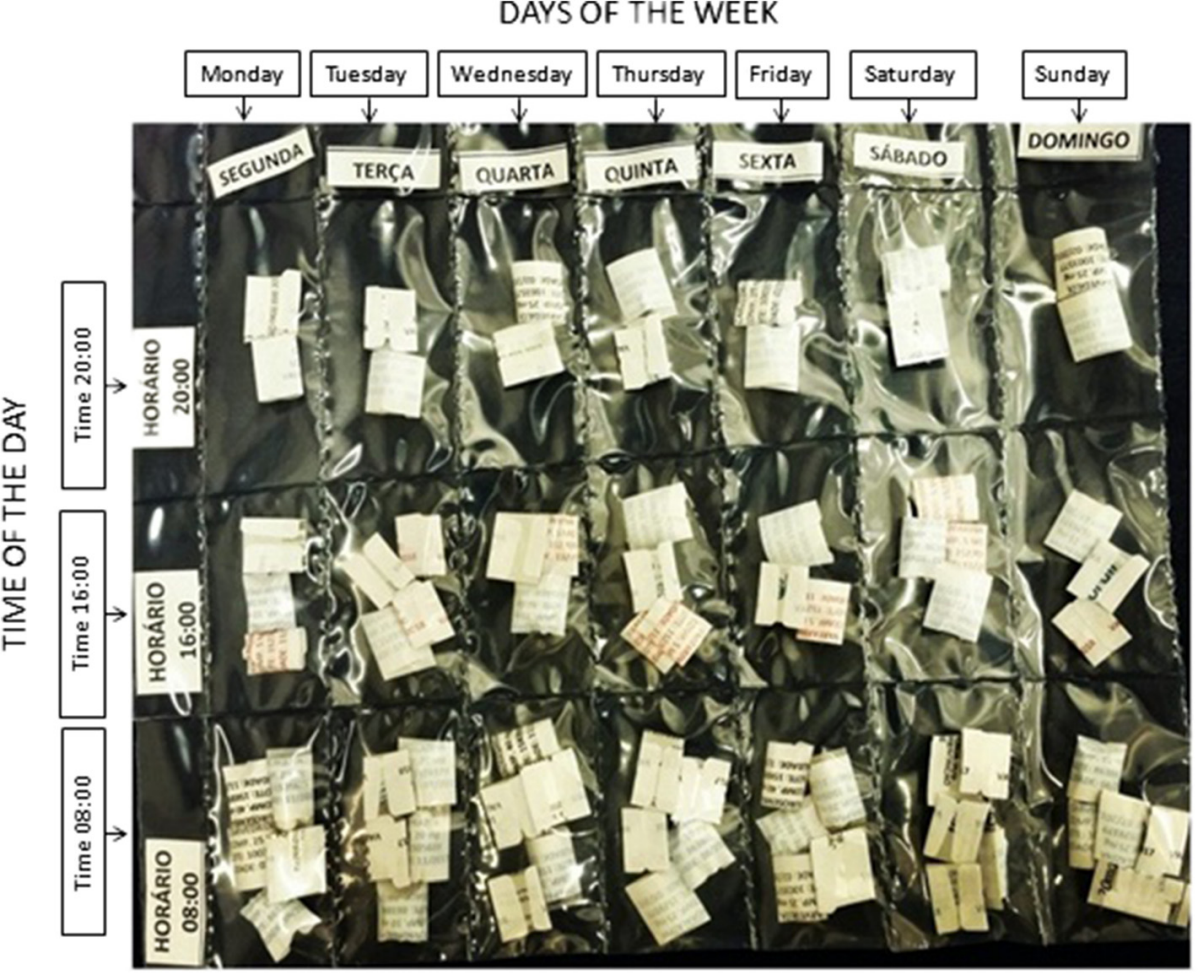
Medication packages

**Fig. 3:**
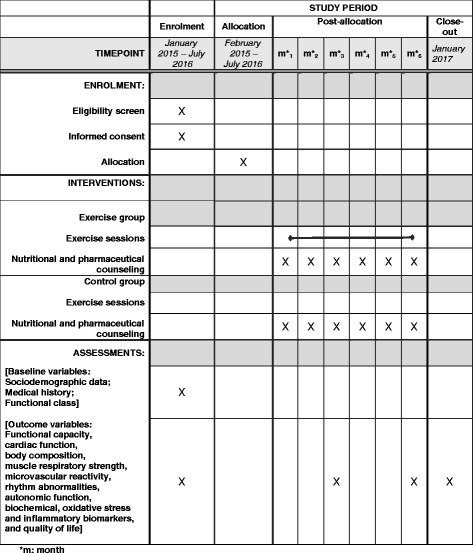
Schedule of enrollment, interventions, and assessments of the PEACH study

Patients of both groups will undergo regular medical appointments with the same cardiologist during the follow-up based on standard medical treatment of the Brazilian consensus on Chagas disease recommendations [3] and with other medical specialties if necessary. In addition, nutritional and pharmaceutical counseling will be provided monthly for both groups. The nutritional counseling will consist of general guidance about healthy eating habits and will include how to reduce saturated fat and include poly and monounsaturated fatty acids, to consume more vitamins, high-fiber carbohydrates, and to reduce the sodium and water intake for patients with HF [16, 17]. Pharmacists will guide patients about medication usage, drug dosage, and compliance. In order to reduce any bias due to medication noncompliance, all patients will receive monthly personalized packages according to the medical prescription. The pills will be organized by the time and days that the pills should be taken (Fig. 2). The schedule of enrollment, interventions, and assessments of the PEACH study is seen in Fig. 3 and the Standard Protocol Items: Recommendations for Interventional Trials (SPIRIT) checklist in Additional file 1.

### Outcomes

These will be functional capacity, cardiac function, body composition, muscle respiratory strength, microvascular reactivity, cardiac rhythm abnormalities, autonomic function, biochemical, oxidative stress and inflammatory biomarkers, and quality of life will be assessed at baseline, 3 months, and 6 months of the study.

#### Cardiopulmonary exercise test

The primary outcome is functional-capacity-based on VO_2_ peak obtained by a CPET using the VO_2000_ gas analyzer (MedGraphics®, St. Paul, MS, USA) with a computerized system Ergo PC Elite (Micromed®, Brasília, Brazil) and a treadmill (Inbramed®, Porto Alegre, Brazil). A blinded evaluator will perform an incremental exercise test using a ramp protocol, tailored to achieve a fatigue limited exercise duration of approximately 8 to 12 minutes. [18]. The workloads will be based on age, gender, height, and weight; adapted to each subject’s physical condition and effort tolerance. Gas and volume calibrations will be executed on the early morning of each test day. Pulmonary gas exchange will be analyzed breath-by-breath and averaged every 10 s. A 12-lead electrocardiogram will monitor heart rhythm during CPET.

The CPET will be limited by symptoms through the subjective fatigue perception scale (Borg modified) ranging from 0 to 10, with 0 representing absence of fatigue and 10 maximum tolerated efforts. The examiner may interrupt the test in case of identification of any harmful hemodynamic response. The recovery phase will be active with walking in a pre-determined velocity of 2 km.h^−1^ and 2 % of inclination.

The following CPET variables will be analyzed: maximum achieved heart hate (HRmax); maximum achieved blood pressure (BPmax); respiratory exchange ratio (VCO_2_/VO_2_); oxygen consumption at peak of exercise (VO_2_ peak); oxygen consumption at anaerobic threshold (VO_2_ AT); oxygen pulse (O_2_ pulse); ventilation slope equivalent to carbon dioxide production (slope VE/VCO_2_); circulatory power (CP); presence of complex ventricular arrhythmias (VA); functional aerobic impairment (FAI); and oxygen uptake efficiency slope (OUES) values.

The VO_2_ peak during exercise will be defined as the greatest value during 30 s before and after maximum effort. The VO_2_ AT will be determined by the point at which expired carbon dioxide increases in a nonlinear fashion relative to the rate of oxygen consumption according to the V-slope method. Ergo PC Elite software will determine the other variables obtained on the CPET.

Before CPET, the rest electrocardiogram will be evaluated to define if the cardiac rhythm is acceptable to perform the exam. The following markers will not be accepted: sustained ventricular tachycardia, second- and third-degree atrioventricular block, atrial or supraventricular tachycardia (more than 100 bpm).

#### Cardiac function

Patients will undergo a standard two-dimensional resting echocardiogram using a phased-array ultrasound system (Vivid 7, General Electric Medical Systems®, Milwaukee, WI, USA) equipped with a M4S transducer. Cardiac dimensions, LV and right ventricular (RV) systolic function, and Doppler measurements will be measured as recommended by the American Society of Echocardiography [19–21]. The biplane Simpson’s method will be used to estimate LVEF. The other studied variables will be as follows: RV peak systolic myocardial velocity (RVS), tricuspid annular plane systolic excursion (TAPSE), RV systolic pressure, peak early (E) and late (A) diastolic filling velocities, E/A ratio, peak, and peak early (E’) diastolic myocardial velocities.

#### Body composition

The anthropometric evaluation will consist of measurements of body weight and height with minimal clothing and without shoes using a calibrated digital scale with coupled stadiometer. A ratio between weight (kg) and squared height (m) will determine body mass index (BMI), an important surrogate of nutritional status [22]. Two circumferences will be taken: the waist circumference at the narrowest waist level and the hip circumference at the largest circumference around the buttocks [23].

A seven-site skinfold thickness protocol including chest, midaxillary, triceps, subscapular, abdomen, suprailiac, and thigh sites will be used to evaluate body composition. Measurements will be taken twice on the right side of the body while standing in a relaxed position (Lange skinfold caliper, Beta Technology Inc., Cambridge, MD, USA). The average of each of these seven skinfold thickness will be summated and used to estimate body composition using the Jackson and Pollock equation [24, 25].

#### Respiratory muscle strength

Respiratory muscle strength will be assessed by maximal inspiratory (MIP) and expiratory pressures (MEP) using a digital pressure manometer connected to a mouthpiece (MVD 3000®, Globalmed, Brazil). Patients will remain in a seated position with a nose clip. They will be requested to make a maximum inspiratory effort at residual volume and a maximum expiratory effort at total lung capacity, sustaining it for 1 to 2 s [26]. Once the operator is satisfied, the maximum value of three maneuvers that vary by less than 20 % will be recorded [27].

#### Microvascular reactivity

A laser speckle contrast imaging system with a laser wavelength of 785 nm (PeriCam PSI system, Perimed, Järfälla, Sweden), coupled to iontophoresis of acetylcholine and sodium nitroprusside, will noninvasively measure real time cutaneous microvascular flow changes in the forearm [28, 29].

For the post-occlusive reactive hyperemia (PORH) test, arterial occlusion will be performed with suprasystolic pressure (50 mmHg above the systolic arterial pressure) using a sphygmomanometer applied to the arm of the subject over 3 min. Peak skin flow will be measured after pressure release.

Images will be analyzed using the manufacturer’s software (PIMSoft, Perimed, Järfälla, Sweden). The measurements of skin blood flow will be divided by the mean arterial pressure to yield the cutaneous vascular conductance (CVC) in arbitrary perfusion units (APU)/mmHg, to avoid interference of blood pressure levels on microvascular flow.

#### Cardiac rhythm abnormalities

Arrhythmias and heart rate variability (HRV) will be evaluated with a 24-h Holter (portable three-channel recorder and analyzer; Cardio Light® and CardioSmart® 5.0, Cardio System, São Paulo, Brazil). Patients will be requested to maintain their normal daily activities during the exam. Standard frequency- and time-domain heart rate variability indexes will be measured and evaluated in patients who do not have artificial pacing or atrial fibrillation rhythm. Only tracings of at least 18 h will be studied. Standard time-domain HRV indices (SDNN: standard deviation of normal-to-normal RR intervals; SDANN: standard deviation of the averages of normal-to-normal RR intervals in all 5-min segments of a 24-h recording; SDNNIDX: mean of the standard deviations of normal-to-normal RR intervals in all 5-min segments of a 24-h recording; rMSSD: root mean square of successive differences; and PNN >50: percentage of differences between adjacent normal-to-normal RR intervals that are greater than 50 ms) and frequency domain (TP: total power; VLFP: very low-frequency power; LFP: low-frequency power; HFP: high-frequency power) will be calculated [30–32].

#### Cardiovascular autonomic function test

The active orthostatic stress test consists of the evaluation of the heart rate and blood pressure response obtained from orthostatic change. The patient will rest for 5 min in the supine position, which will be followed by a quick stand-up position (3 to 5 s). The electrocardiogram will be digitally recorded (ErgoMet 13 V1.0.3.0 HW Heart Ware, Porto Alegre, Brazil) 10 s before the maneuver and will last until 40 s after. The RR intervals will be measured throughout the test period. The baseline average heart rate will be based on the 10 RR intervals immediately preceding the maneuver. The maximum RR at rest over the minimum RR after standup ratio will be calculated (index max:min RR) [33]. The blood pressure will be measured after 5 min at rest and the systolic blood pressure at 5 s after standing with diastolic blood pressure collected within 5 s after recording systolic blood pressure immediately after standing up to evaluate postural hypotension. Patients with artificial pacing or atrial fibrillation rhythm during the exam will not be evaluated.

#### Laboratorial biomarkers

A laboratory accredited by the College of American Pathologists will perform biochemical measurements. Total cholesterol, high-density lipoprotein (HDL) cholesterol, triacylglycerol, glucose, glycated hemoglobin, and the N-terminal of the prohormone brain natriuretic peptide (NT-proBNP) will be measured using Siemens Dimension® reagent cartridge with an intra- and inter-assay coefficient of variation (CV) <5 %. The Friedewald equation, based on the triacylglycerol measures, will be used to determine low-density lipoprotein (LDL) cholesterol and very low-density lipoprotein (VLDL) cholesterol concentrations [34].

Cytokine serum levels will be measured accordingly to the manufacturer’s instructions (EBioscience, San Diego, CA, USA). Antibodies specific for interferon gamma (IFN-γ), tumor necrosis factor (TNF), interleukin-beta 1 (IL-1β), interleukin-10 (IL-10), interleukin-4 (IL-4), interleukin-8 (IL-8), or monocyte chemotactic protein 1 (MCP-1) will be coated onto the 96-well ELISA microplate overnight. Washing solution will be added to each well three times. Standards and unknown samples will be pipetted into these wells and will be incubated for 2 h. After washing, a biotinylated (detection) antibody specific for the described cytokines will be added and incubated for 1 h. After washing, streptavidin-horseradish peroxidase will be added. After incubation for 30 min and washing to remove all unbound enzyme, color development solution will be added. Then, the plates will be read using a microplate reader (SpectraMax 190, Molecular Devices, Sunnyvale, CA, USA) at 450 nm. Oxidative stress will be accessed by two different methodologies: detection of serum carbonylated proteins and reduced/oxidized glutathione ratio. The oxidative modified serum proteins will be detected after derivatization with 2,4-dinitrophenylhydrazine, through generation of dinitrophenylhydrazone, which will be analyzed in a spectrophotometer at 380 nm [35]. The reduced and oxidized glutathione pool will be determined in patients’ sera using DetectX® Glutathione Fluorescent Detection Kit (Arbor Assays, Ann Arbor, MI, USA) as recommended by the manufacturer.

#### Quality of life

The short-form version of the SF-36 Health Survey [36] and the Minnesota Living with Heart Failure Questionnaire [37] will be used to assess quality of life, all of them previously translated and validated in Portuguese.

The SF-36 consists of 36 questions in eight different domains: general health, physical functioning, social functioning, mental health, physical role, emotional role, bodily pain, and vitality. Each of these dimensions range from 0 (worst possible health state) to 100 (best possible health state).

The Minnesota Living with Heart Failure Questionnaire has 21 questions about how the heart disease influences the lifestyle related to physical, psychological, and social areas. Each question’s responses range from 0 (none) to 5 (very much) and the maximum score is 105. In this questionnaire, lower scores mean better quality of life.

### Sample size

Considering a difference in peak oxygen intake of 2.9 ml.kg^−1^.min^−1^ with a standard deviation of 2.0 ml.kg^−1^.min^−1^ [38], assuming an *α* = 0.05 and *β* = 0.20, and increasing the sample size by 50 % accounting for losses to follow-up, a total of 30 patients (15 in the control group and 15 in the exercise group) will be included.

### Randomization

A sequence will be computer-generated to randomly allocate 30 patients into two groups in a 1:1 ratio (WinPepi version 11). The sequence will be generated in blocks and by strata of CHD classification (B2 and C) by a single researcher not involved in recruitment. Opaque envelopes will be filled in sequentially to either control or exercise group. Block size will be blinded from investigators involved in patients’ recruitment.

### Blinding

Given that exercise implies a behavioral intervention, it is not feasible to blind the patients. However, the evaluators will be blinded to the primary endpoint obtained by the CPET and the following secondary endpoints: microvascular reactivity, cardiac rhythm abnormalities, cardiovascular autonomic function test, and laboratorial biomarkers. A blinded researcher will perform all data analysis.

### Interim analysis and stopping rules

Three interim analyses are planned. The first will be conducted when the tenth volunteer completes 3 months of follow-up, the second when the twentieth volunteer completes 3 months of follow-up and the third when the last volunteer completes 3 months of follow-up.

Trial interruption for ethical reasons due to either positive or negative results exceeding expectations may be recommended by an independent committee. The pre-specified stopping rule is a difference of 50 % in VO_2_ peak between groups, serious adverse events twice as frequent in one of the groups as cardiovascular death, acute myocardial infarction, unstable angina, cardiopulmonary arrest, malignant ventricular arrhythmias, decompensated HF, and stroke. All these estimates should have a significance level of 0.01 or less in any of the interim analyses.

### Statistical analysis

Descriptive analysis will consist of mean and standard deviation for continuous variables and percentage for categorical variables. Skewness and Kurtosis testing will be performed to assess the normality of data which will be log-transformed in case of skewed distribution. Variables that can change prognosis of the disease will be compared at baseline in relation to the exercise and control groups. Longitudinal effects of exercise on primary and secondary outcomes will be evaluated through linear mixed models (LMM), which correlate with repeated measures over the time. LMM is an intention-to-treat analysis as it includes all observations of each one of the patients regardless of losses to follow-up or noncompliance to exercise protocol. The longitudinal analysis will be made by the treatment × time interaction, which estimates the rate of changes in the outcomes. Residual plots of all models will be examined and the likelihood-ratio test will be used to compare and select random intercept or random slope models.

The REDCap software will be used for data management and the data analysis will be conducted by Stata 13.0 software. Statistical significance will be set at *p* < 0.05 for all analyses.

## Discussion

Despite major advances in cardiovascular therapies, CHD still stands as an important cause of premature death in Latin America. Although the number of new cases of Chagas disease has decreased steadily since the late 1990s, many chronic cases are still part of routine care in public hospitals where patients with lower income have access to treatment. Moreover, decreased barriers to international travel and migration has led to an increase in migration of patients from Chagas disease-affected areas to nonendemic countries of North America and Europe. This globalization phenomenon transformed Chagas disease into a global medical challenge [39, 40].

CHD treatment is based on trials that studied the effect of different drugs on ventricular dysfunction, survival, and quality of life in patients with cardiomyopathies from other etiologies [4, 41]. However, CHD has a specific autonomic imbalance, a different pattern of myocardial fibrosis associated with an inflammatory milieu generated by parasite and host defenses, an increased risk of complex arrhythmias and a known worse prognosis than cardiomyopathies from other etiologies [42, 43].

Exercise training is becoming an important strategy in the treatment of patients with cardiac diseases. Numerous studies demonstrate that regular exercise is safe and associated with substantial benefits in patients with cardiovascular disease, mainly from ischemic etiology. Clinical adaptations to exercise training include improvements in functional capacity, enhancements in cardiac and vascular function, autonomic nervous system modulation, decreases in oxidative stress and low-grade inflammation, and improvements in lipid and glucose profiles [44, 45].

Despite these well-established benefits of exercise training in cardiac patients, there are few studies analyzing its effects on patients with CHD. Currently, only one randomized clinical trial [11] including 40 patients with CHD showed that exercise induced improvements in functional capacity and health-related quality of life. This study demonstrates that exercise is feasible, effective, and safe in patients with CHD but with restrictions as the indirect measurement of VO_2_ peak, the short-term follow-up and the inclusion of patients in the early stages of CHD, which preclude a definite conclusion about the effects of exercise training in this population.

In the PEACH study, we will try to fill this knowledge gap and address the issue of whether patients with CHD have the same benefits promoted by exercise training in patients with cardiomyopathies from other etiologies. We hypothesize that exercise training will be safe and promote improvements in functional capacity and quality of life, as previously demonstrated by Lima et al. [11] and Fialho et al. [12] in a different sample of patients with CHD. Since an exacerbated inflammatory response is an important mechanism involved in the development of CHD [46] and because several studies have been demonstrating an important anti-inflammatory property of exercise training [47], a decrease in the serum levels of pro-inflammatory cytokines and an increase in the serum levels of anti-inflammatory cytokines in patients with CHD in the exercise group is expected. Although enhancements in autonomic function as results of exercise training are present in studies with other cardiomyopathies [48], a recent article did not confirm this finding in patients with CHD [30]. We also hypothesize that cardiac function will improve by an increase in LVEF and an improvement in diastolic function, as seen in coronary artery disease [49], and that exercise will improve body composition, respiratory strength, microvascular reactivity, and oxidative stress agents based on results from studies evaluating exercise in non-CHD patients [50, 51].

The benefits of exercise training that will be described by this study will set a new treatment strategy for CHD patients and that this strategy could be routinely included in clinical practice.

## Trial status

Participants are currently being recruited.

## Additional file

Additional file 1: SPIRIT checklist. (DOC 121 kb)

## Abbreviations

A: Late diastolic filling velocity
APU: Arbitrary perfusion units
BMI: Body mass index
BPmax: Maximum achieved blood pressure
CHD: Chagas heart disease
CP: Circulatory power
CPET: Cardiopulmonary exercise test
CR: Cardiac rehabilitation
CV: Coefficient of variation
CVC: Cutaneous vascular conductance
E: Early diastolic filling velocity
E’: Peak early diastolic myocardial velocity
EF: Ejection fraction
ELISA: Enzyme-linked immunosorbent assay
FAI: Functional aerobic impairment
HDL: High-density lipoprotein
HF: Heart failure
HFP: High-frequency power
HRmax: Maximum achieved heart hate
HRV: Heart rate variability
IFN-γ: Interferon gamma
IL-10: Interleukin 10
IL-1β: Interleukin beta 1
IL-4: Interleukin 4
IL-8: Interleukin 8
INI: Evandro Chagas National Institute of Infectious Diseases
IPAQ-SF: International Physical Activity Questionnaire-Short Form
LDL: Low-density lipoprotein
LFP: Low-frequency power
LMM: Linear mixed models
LV: Left ventricular
LVEF: Left ventricular ejection fraction
MCP-1: Monocyte chemotactic protein 1
MEP: Maximal expiratory pressure
MIP: Maximal inspiratory pressure
NT-proBNP: N-terminal of the prohormone brain natriuretic peptide
O_2_ pulse: Oxygen pulse
OUES: Oxygen uptake efficiency slope
PEACH study: Exercise program in Chagas heart disease
PNN >50: Percentage of differences between adjacent normal-to-normal RR intervals that are greater than 50 ms
PORH: Post-occlusive reactive hyperemia
rMSSD: Root mean square of successive differences
RV: Right ventricular
RVS: Right ventricular peak systolic myocardial velocity
S: Peak systolic myocardial velocity
SDANN: Standard deviation of the averages of normal-to-normal RR intervals
SDNN: Standard deviation of normal-to-normal RR intervals
SDNNIDX: Mean of the standard deviations of normal-to-normal RR intervals
slope VE/VCO_2_: Ventilation slope equivalent to carbon dioxide production
TAPSE: Tricuspid annular plane systolic excursion
TNF: Tumor necrosis factor
TP: Total power
VA: Presence of complex ventricular arrhythmias
VCO_2_/VO_2_: Respiratory exchange ratio
VLDL: Very low-density lipoprotein
VLFP: Very low-frequency power
VO_2_ AT: Oxygen consumption at anaerobic threshold
VO_2_ peak: Oxygen consumption at peak of exercise
